# Direct spread of thyroid follicular carcinoma to the parotid gland and the internal jugular vein: a case report

**DOI:** 10.1186/1752-1947-2-297

**Published:** 2008-09-09

**Authors:** Ahmed Alzaraa, Jason Stone, Glyn Williams, Irfan Ahmed, Mohammed Quraishi

**Affiliations:** 1 Department of Otolaryngology, Doncaster Royal Infirmary, Doncaster, UK; 2 Department of Histopathology, Doncaster Royal Infirmary, Doncaster, UK; 3 Department of Radiology, Doncaster Royal Infirmary, Doncaster, UK

## Abstract

**Introduction:**

The parotid gland and the great cervical veins are very rarely involved in a metastatic thyroid cancer.

**Case presentation:**

We report an interesting case of an unusual metastasis of a thyroid follicular carcinoma including the histopathological and radiological findings. A woman was seen in the otolaryngology clinic with a mass at the angle of the left side of her jaw. Clinical examination and investigations confirmed a thyroid follicular carcinoma with metastases to the parotid gland and the internal jugular vein.

**Conclusion:**

This is an educational case which highlights the importance of close communication between clinicians, histopathologists and radiologists to ensure that such rare cases are not missed.

## Introduction

Thyroid carcinoma sometimes shows a microscopic vascular invasion, but gross angioinvasion with intraluminal thrombosis is extremely rare. Very few cases about metastasis of thyroid cancer to the internal jugular vein, and fewer cases about metastasis to the parotid gland have been separately reported. Our patient has both these organs involved by direct spread from a thyroid follicular carcinoma.

## Case presentation

A 78-year-old woman was seen in the otolaryngology clinic in June 2006 with a painless swelling at the angle of the left side of her jaw which had been present for 9 months. The mass had slightly increased in size over this period. The patient had tinnitus but no other complaints. Her weight was stable. Clinical examination revealed a smooth, soft lesion in the tail of the left parotid gland. There was no cervical lymphadenopathy. The ears, nose and throat were normal and the facial nerve was intact.

**Figure 1:**
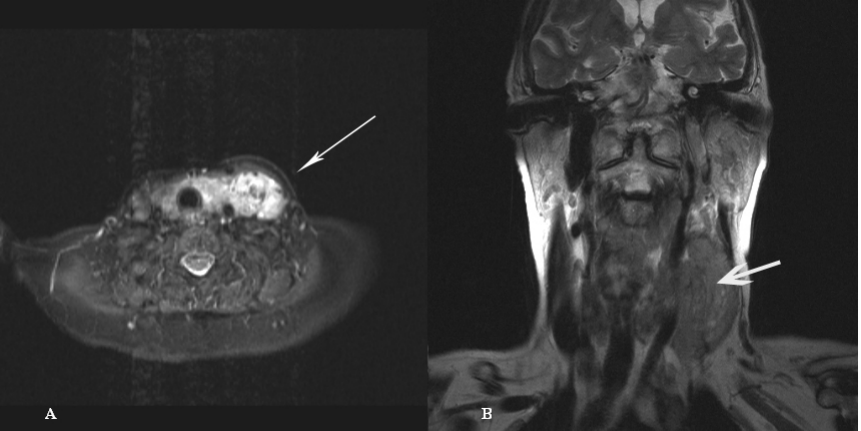
Coronal T2 weighted image **(A) **and STIR sequence **(B) **showing left thyroid tumour extending directly into the left internal jugular vein.

**Figure 2:**
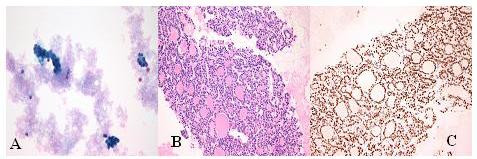
Parotid aspirate (A & B) showing thyroid follicular cells. Nucleus positive immunohistochemistry for Thyroid Transcription Factor-1 confirms thyroid origin (C).

Ultrasound of the neck showed swellings in the left parotid gland and the left thyroid lobe. Fine needle aspiration (FNA) of the left parotid gland showed thyroid follicular cells. A magnetic resonance imaging (MRI) scan of the neck confirmed both soft tissue masses with extensive thrombosis of the left internal jugular vein contiguous with the primary tumour (Figure 1A and 1B). A computed tomography (CT) scan of the chest was normal. Subsequent FNA of the left thyroid lobe and the internal jugular vein (IJV) revealed thyroid follicular cells similar to those seen in the first FNA. The cells were positive for thyroglobulin and thyroid transcription factor 1 and negative for chromogranin and synaptophysin on immunohistochemistry, confirming the diagnosis of a thyroid follicular carcinoma (Figure 2A, B and 2C). Although the patient was not fit for aggressive surgery, she was given two courses of radioiodine. An uptake scan performed approximately 14 months after diagnosis (6 weeks after her last course of radioiodine) showed no further significant iodine uptake. At that time she was clinically well with no palpable residual or recurrent disease. She is still on routine follow-up in the oncology clinic.

## Discussion

Invasion of the parotid gland and the great cervical veins from a thyroid cancer is extremely rare, and is mostly detected at autopsy [1-3]. Both of these organs were involved in our patient following direct spread from a thyroid follicular carcinoma. Two general types of metastases should be distinguished in metastatic salivary gland tumours: regional metastases (head and neck) and distant metastases [4].

Involvement of the parotid gland by invasion or spread by metastases from malignant tumours in the head and neck is uncommon, with the exception of melanoma of the temple, scalp and ear, and anaplastic squamous cell carcinoma of the ear and ear canal [5]. Seifort et al. [4] reported three cases of a metastatic thyroid cancer to the parotid in their analysis of 108 cases of secondary metastases to salivary glands. Another case was found by the Pack Medical Group among 81 cases of parotid gland involvement as a secondary extension of malignant tumours [5]. It is more common for the parotid gland to be involved as an incidental part of a generalized metastatic disease rather than a site of isolated metastasis. This gland contains 20 to 30 lymph follicles and lymph nodes connected with a rich interlacing network of lymph vessels. Lymph entrance to the gland may be direct, without involvement of the paraglandular lymph nodes, may be secondarily deposited from paraglandular lymph nodes, or may contaminate the system by retrograde extension from massive metastases in the neck [6]. Clinically and pathologically, secondary spread to the parotid manifests itself as a primary salivary gland tumour that may mislead clinicians, radiologists and pathologists [6].

The cytological recognition of a thyroid metastasis to different body sites may pose a diagnostic difficulty, especially when a thyroid cancer presents initially at the metastatic site. Immunohistochemical thyroglobulin positivity is a useful tool in distinguishing between a thyroid primary and other metastatic lesions, as this marker is specific for thyroid tumours [6]. Once the parotid has become a focus of metastasis in malignant tumours of the head and neck, the prognosis is grave [5].

Thyroid carcinoma sometimes shows a microscopic vascular invasion, but rarely causes tumour thrombus in the internal jugular vein or the great veins of the neck [7]. The tumour thrombus is the result of a tumour extension from the thyroid gland to the IJV or the result of occult vascular spreading. The most common clinical manifestation is a dilated vein. Findings on neck palpation are usually non-specific and may reveal oedema and tenderness of the sternocleidomastoid muscle and the surrounding soft tissues [7].

The primary management of an advanced disease with vascular invasion would be radical surgery to remove a macroscopic disease. This is followed by high-dose radioiodine ablative therapy with or without external beam radiotherapy and suppression of thyroid stimulating hormone [3]. The role of chemotherapy in these cases remains unproven.

## Conclusion

This rare case of a thyroid follicular carcinoma presenting as a metastasis in the parotid gland serves to highlight the importance of remaining clinically vigilant to the possibility that a salivary gland lesion may be a metastasis from another site. The necessity of communication between clinicians, histopathologists and radiologists is also well illustrated by this case. This very rare presentation of a thyroid follicular carcinoma could easily have been reported incorrectly as benign thyroid follicular cells if there was poor communication and the reporting pathologist was not made aware that the initial aspirate was from the parotid gland and not from the thyroid gland.

## Abbreviations

CT: computed tomography; FNA: fine needle aspiration; IJV: internal jugular vein; MRI: magnetic resonance imaging; TTF-1: Thyroid Transcription Factor 1.

## Competing interests

The authors declare that they have no competing interests.

## Consent

Written informed consent was obtained from the patient for publication of this case report and accompanying images. A copy of the written consent is available for review by the Editor-in-Chief of this journal.

## Authors' contributions

AA performed the literature search, and drafted and revised the manuscript. JS evaluated the histological slides. GW evaluated the radiological images. IA assisted with the literature search. MQ edited the manuscript. All authors have read and approved the final manuscript.
